# Ion Mobility Coupled to a Time-of-Flight Mass Analyzer Combined With Fragment Intensity Predictions Improves Identification of Classical Bioactive Peptides and Small Open Reading Frame-Encoded Peptides

**DOI:** 10.3389/fcell.2021.720570

**Published:** 2021-09-17

**Authors:** Marlies K. R. Peeters, Geert Baggerman, Ralf Gabriels, Elise Pepermans, Gerben Menschaert, Kurt Boonen

**Affiliations:** ^1^BioBix, Department of Data Analysis and Mathematical Modelling, Ghent University, Ghent, Belgium; ^2^Centre for Proteomics, University of Antwerp, Antwerp, Belgium; ^3^Unit Environmental Risk and Health, Flemish Institute for Technological Research, Mol, Belgium; ^4^Department of Biomolecular Medicine, Ghent University, Ghent, Belgium; ^5^VIB-UGent Center for Medical Biotechnology, Flanders Institute for Biotechnology, Ghent, Belgium; ^6^OHMX.bio, Ghent, Belgium

**Keywords:** peptidomics, proteogenomics analysis, neuropeptide, sORF-encoded polypeptide (SEP), micropeptide, spectral intensity prediction, non-coding, timsTOF Pro mass spectrometry

## Abstract

Bioactive peptides exhibit key roles in a wide variety of complex processes, such as regulation of body weight, learning, aging, and innate immune response. Next to the classical bioactive peptides, emerging from larger precursor proteins by specific proteolytic processing, a new class of peptides originating from small open reading frames (sORFs) have been recognized as important biological regulators. But their intrinsic properties, specific expression pattern and location on presumed non-coding regions have hindered the full characterization of the repertoire of bioactive peptides, despite their predominant role in various pathways. Although the development of peptidomics has offered the opportunity to study these peptides *in vivo*, it remains challenging to identify the full peptidome as the lack of cleavage enzyme specification and large search space complicates conventional database search approaches. In this study, we introduce a proteogenomics methodology using a new type of mass spectrometry instrument and the implementation of machine learning tools toward improved identification of potential bioactive peptides in the mouse brain. The application of trapped ion mobility spectrometry (tims) coupled to a time-of-flight mass analyzer (TOF) offers improved sensitivity, an enhanced peptide coverage, reduction in chemical noise and the reduced occurrence of chimeric spectra. Subsequent machine learning tools MS^2^PIP, predicting fragment ion intensities and DeepLC, predicting retention times, improve the database searching based on a large and comprehensive custom database containing both sORFs and alternative ORFs. Finally, the identification of peptides is further enhanced by applying the post-processing semi-supervised learning tool Percolator. Applying this workflow, the first peptidomics workflow combined with spectral intensity and retention time predictions, we identified a total of 167 predicted sORF-encoded peptides, of which 48 originating from presumed non-coding locations, next to 401 peptides from known neuropeptide precursors, linked to 66 annotated bioactive neuropeptides from within 22 different families. Additional PEAKS analysis expanded the pool of SEPs on presumed non-coding locations to 84, while an additional 204 peptides completed the list of peptides from neuropeptide precursors. Altogether, this study provides insights into a new robust pipeline that fuses technological advancements from different fields ensuring an improved coverage of the neuropeptidome in the mouse brain.

## Introduction

The term “peptidomics” was first used two decades ago to describe a quantitative and qualitative analysis of the endogenous peptide pool in biological samples (Clynen et al., 2001; Schulz-Knappe et al., 2001; Verhaert et al., 2001; Baggerman et al., 2002). Since then, it has evolved from a new promising “omics” field into a successful method in a wide variety of research areas such as drug and biomarker discovery (Gelman et al., 2013; Hou et al., 2020) along with other clinical applications (Kim et al., 2012; Ghezellou et al., 2021; Melby et al., 2021). The subfield of neuropeptidomics comprises the efforts in characterizing the full repertoire of neuropeptides in the brain or nervous system (Svensson et al., 2007; Gelman and Fricker, 2010; Le et al., 2013) and has led to the identification of several bioactive peptides exerting key roles in complex processes, such as regulation of body weight, learning, aging, and innate immune response (Gelman and Fricker, 2010; Budamgunta et al., 2018). While most established bioactive neuropeptides are cleaved from larger precursor proteins and further modified through the secretory pathway (Gelman and Fricker, 2010; Hayakawa et al., 2019) an emerging field in neuropeptidomics is the study of proteins smaller than 100 amino acids directly translated from open reading frames (Budamgunta et al., 2018). The coding potential of these small open reading frames (sORF)-encoded peptides (SEPs) has been a point of dispute for years, but advances in high-throughput methods and integration of several datatypes (reviewed in Peeters and Menschaert, 2020) has demonstrated their potential as biological regulators. Recently, a peptide encoded in the 5’ untranslated region (UTR) of the calcitonin gene-related peptide (CGRP/*Calca*) has been reported to trigger pain-associated behavioral responses *in vivo*, emphasizing a role for upstream open reading frame (uORF) translation in nociceptor plasticity (Barragan-iglesias et al., 2021). Besides uORFs, sORF-encoded peptides translated from originally “non-coding” RNAs are another category of peptides reported with functions in important processes like inflammation and metabolism (Chen et al., 2021). For example, the long non-coding RNA (lncRNA) *Aw112010* harbors a peptide vital to the mucosal immunity (Jackson et al., 2018) where the peptide produced from LINC00493 interacts with mitochondrial proteins (Wang et al., 2021). Another example is Nobody, a recently characterized human microprotein involved in the mRNA decapping machinery, translated from a transcript originally annotated as non-coding (D’Lima et al., 2017) and also identified in mouse (Budamgunta et al., 2018).

The identification of sORFs and their encoded peptides benefits greatly of the combination of multiple techniques (Peeters and Menschaert, 2020). A broad set of computational approaches applied to ribosome profiling data, the sequencing technique capturing the translational landscape at single-nucleotide resolution (McGlincy and Ingolia, 2017), has been used to predict thousands of sORFs across all species (Pueyo et al., 2016; Fabre et al., 2021). However, the true existence of these peptides can be validated only by mass spectrometry (MS)-based technologies. Although the latter is essential for confirmation of the translation and for in-depth functionalization, it remains challenging due to intrinsic characteristics of the said peptides (Wang et al., 2021) such as their specific translation patterns (Budamgunta et al., 2018; Peng et al., 2020) and low copy numbers. The presumable time- and cell specific translation of sORF-encoded peptides (Hollerer et al., 2018) together with their short half-life generally results in an overall low abundance in biological samples, necessitating the reduction of the sample complexity by either enrichment for small peptides or depletion of larger complexes (Petruschke et al., 2020). A broad selection of separation techniques has been applied in several optimized workflows (reviewed in Peng et al., 2020; Fabre et al., 2021). For example, Bartel et al. (2020) increased the identification rate by applying an gel filtration enrichment based on a column coated with small-pore sized solid-phase material, while Kaulich et al. (2020) evaluated separation with SDS-PAGE gels followed by different staining methods. Besides the low abundance, the MS-based identification of the digested SEPs is further impeded by their small size, possibly limiting the number of detectable tryptic peptides using bottom-up proteomic approaches (Orr et al., 2020). Efforts in optimizing the distinct steps of the MS-based workflow for robust SEP detection are ongoing but a digest with trypsin prior to the data-dependent acquisition during MS analysis has become the most common method for general SEP identification (Fabre et al., 2021). Due to the highly variable physicochemical characteristics of these peptides such as different hydrophobicity (Piovesana et al., 2020), a variety of workflows can result in distinct sets of SEPs with specific biochemical properties related to the applied method. As such, a mixture of workflows and alternative techniques, such as diverse collision energy leading to different types of fragmentation spectra (Peng et al., 2020; Fabre et al., 2021) or other digestion methods resulting in a distinct pool of peptides (Bartel et al., 2020), could yield new identifications.

Neuropeptidomics aims to characterize the full neuropeptidome, including SEPs besides other endogenous peptides. Apart from the challenges described above, additional factors further complicate the comprehensive identification of the total bioactive peptide pool. Contrary to proteomics methods, (neuro)peptidomics aims to study peptides in their naturally occurring state (Maes et al., 2019), usually omitting the use of a cleavage enzyme during sample preparation. The high abundance of post-translational modifications (PTMs) in bioactive peptides, often necessary for their biological function (Secher et al., 2016), combined with the lack of knowledge of specific cleavage pattern that has produced the endogenous peptides from their precursor, strongly expands the search space in the peptide to spectrum matching process (Menschaert et al., 2010; Cerrato et al., 2020). The explosion of possible candidates during database searching decreases the sensitivity, thereby increasing the risk for false positives (Bouwmeester et al., 2020; Verbruggen et al., 2021). Hayakawa and colleagues (Hayakawa et al., 2019) tried to circumvent this issue by performing a selective extraction for neuropeptides and searching MALDI MS/MS data against a reduced protein database, only considering *in silico* predicted neuropeptides. Next to that, *de novo* search approaches could potentially offer a solution for more robust searches taking the highly variable PTMs into account (Romanova and Sweedler, 2015; Maes et al., 2019). Proper bioactive peptide identification is further complicated by the lack of digestion leading to a pool of peptides without the regular patterns of typical tryptic peptides with a basic amino acid at the C-terminus that facilitates ionization and fragmentation (Menschaert et al., 2010; Maes et al., 2019). Consequently, mature endogenous peptides often possess unfavorable ionization properties, like multiple internal basic residues (Tabb et al., 2004) and charge heterogeneity (Maes et al., 2019), generating less informative fragmentation patterns and lower quality spectra. Therefore, the number of spectra per individual endogenous peptide is prone to be low, emphasizing the demand for an alternative validation method to improve neuropeptidomics identifications.

Recent advances in machine learning tools and widespread use of high throughput techniques provides a massive amount of data as a source to develop tools for every step in MS-based workflows (Bouwmeester et al., 2020). For example, the post-processing tool Percolator (Käll et al., 2007; Halloran and Rocke, 2018) integrates several features into a semi-supervised learning algorithm to improve the distinction between true and false peptide-spectrum matches. Next to that, spectrum intensity predictors, such as MS^2^PIP (Degroeve et al., 2015; Gabriels et al., 2019) and Prosit (Gessulat et al., 2019) are new models that incorporate fragment ion intensities predictions as additional features next to the standard m/z ratio during spectral library searching to increase the resolution of the identification, even in challenging workflows such as proteogenomics (Verbruggen et al., 2021). Despite the great promise of these tools, only a limited number have been integrated in common workflows (Bouwmeester et al., 2020).

In this study, we introduce a two-step methodology combining firstly a trapped ion mobility spectrometry coupled to a time-of-flight mass analyzer (timsTOF) to generate the highest quality MSMS spectra, secondly the MS^2^ReScore (Silva et al., 2019) application leading to an improved identification of potential bioactive peptides in different regions of the mouse brain. timsTOF offers an enhanced peptide coverage and reduction in chemical noise (Lubeck et al., 2018) because of its extra dimension of separation (Meier et al., 2018), previously described in different applications in the field of clinical proteomics (Azkargorta et al., 2020; Macron et al., 2020; Hamada et al., 2021; Liu et al., 2021). The MS^2^ReScore tool, including MS^2^PIP that predicts the fragment ion intensity as an additional feature, DeepLC predicting retention times (Bouwmeester et al., 2021) and the post-processing tool Percolator, further boost the yield of identified peptides. Additional PEAKS analysis further expanded the pool of neuropeptides and SEPs on non-coding regions. In conclusion, this study fuses technological advances of different fields leading to an improved coverage of the neuropeptidome in the mouse brain.

## Materials and Methods

### Sample Collection

Experimental procedures and protocols were performed following European Directive 86/609/EEC Welfare and Treatment of Animals and were approved by the local ethical committee (2019-50, University of Antwerp, Belgium). Brains from early postnatal Swiss CD1 mice were dissected and flash frozen: one brain from a postnatal day 1 (P1) mouse (in its entirety) and one brain from a P5 mouse that was separated in four parts (after removal of cortex and cerebellum the remaining part was divided by a coronal section in an anterior and a posterior part). After storage at −80°C an icecold mixture of methanol:water:acetic acid (90:9:1) was added and samples were stirred 15 min at full speed in a Thermomixer at 4°C. Samples were centrifuged 15 min at 16,000 *g* after which the supernatants was dried in a speedvac vacuum concentrator. The dried pellet was resuspended in 1% acetonitrile, 0.1% formic acid after which the sample was cleaned up using C18 reversed phase spin columns (Thermo Fisher Scientific) according to manufacturer’s protocol.

### Liquid Chromatography Mass Spectrometry

The sample was dissolved in 10 μl of 6% ACN and 0.1% FA and separated on a ACQUITY UPLC M-Class System (Waters), fitted with a nanoEase^TM^ M/Z Symmetry C18 trap column (100 Å, 5 μm, 180 μm × 20 mm) and a nanoEase^TM^ M/Z HSS C18 T3 Column (100 Å, 1.8 μm, 75 μm × 250 mm, both from Waters). The sample was loaded onto the trap column in 2 min at 5 μl/min in 94% solvent A 6% solvent B (solvent A is 0.1% FA in 18.2 MOhm^∗^cm water (MilliQ), solvent B 0.1% FA in 80% ACN). The flow over the main column was 0.4 μl/min and the column was heated to 40°C. After an isocratic flow of 4 min at 6% B, the concentration of B increased in 36 min to 50% B, to 94% B in 4 min. After again an isocratic flow of 4 min at 94% B, the concentration of B decreased in 4 min to 6% which was followed by 15 of equilibration at 6%.

The column was online with a timsTOF Pro operating in positive ion mode, coupled with a CaptiveSpray ion source (both from Bruker Daltonik GmbH, Bremen). The timsTOF Pro was calibrated according to the manufacturer’s guidelines. The temperature of the ion transfer capillary was 180°C. The Parallel Accumulation–Serial Fragmentation DDA method was used to select precursor ions for fragmentation with 1 TIMS-MS scan and 10 PASEF MS/MS scans, as described by Meier et al. (2018). The TIMS-MS survey scan was acquired between 0.70 and 1.45 Vs/cm^2^ and 100–1,700 *m/z* with a ramp time of 166 ms. The 10 PASEF scans contained on average 12 MS/MS scans per PASEF scan with a collision energy of 10 eV. Precursors with 1–6 charges were selected with the target value value set to 20,000 a.u and intensity threshold to 2,500 a.u. Precursors were dynamically excluded for 0.4 s. The timsTOF Pro was controlled by the OtofControl 5.1 software (Bruker Daltonik GmbH). Ten PASEF scans can contain up to 12 MS/MS scans per PASEF scan. Raw data was analyzed with the DataAnalysis 5.1 software (Bruker Daltonik). The resulting d folder obtained from the Bruker software for each run individually was uploaded into the alphatims tool^1^ (Willems and Mann, 2021) (run via command line) to create a centroid mgf file for further processing. The mass spectrometry data have been deposited to ProteomeXchange Consortium via the PRIDE partner repository with the dataset identifier PXD026584.

### Peptide Identification: Database Construction and Searching

A custom proteogenomic search database was constructed combining the known *Mus musculus* UniProt reference proteome (downloaded at 8/10/2020) and an alternative proteome based on the sORFs.org method (Olexiouk et al., 2016, 2018) and the OpenProt repository (Brunet et al., 2021). First, seven publicly available ribosome profiling datasets from mouse brain tissues were downloaded from NCBI Gene Expression Omnibus (GEO) [GSE140565, GSE143330, and GSE143331 (Shah et al., 2020), GSE94982, GSE112223 (Gerashchenko et al., 2021), GSE119681 (Zhao et al., 2019), GSE51424 (Gonzalez et al., 2014), and GSE74683 (Laguesse et al., 2015)]. These datasets were subjected to the previously published sORF prediction pipeline (Olexiouk et al., 2018) with minor code modifications (available upon request). The sORF predictions of all datasets were combined into one FASTA file, where only the longest predicted sORF sequence for each stop position was considered and sORF predictions only spanning over a single exon of annotated protein coding genes removed. Additionally, the combined FASTA file was deduplicated and analyzed by an in-house scripting module (written in Python 3.7, available upon request) to exclude identical overlapping sequences with the reference proteome and to construct compatible headers. Next, the alternative proteome was downloaded from OpenProt (Brunet et al., 2021) (on 25/6/2020, release 1.5, only containing altprots and isoprots) and compared against the sORFs.org predictions for overlapping sequences with the same previously mentioned module. Finally, all different parts were concatenated with the cRAP database (downloaded on September 16, 2020) to account for common contaminants in proteomics samples, reverse sequences were added as decoys and the resulting fasta file was used in subsequently database searches in SearchGUI (v4.0.32) (Barsnes and Vaudel, 2018). MSGF + was chosen as the search engine (Kim and Pevzner, 2014), setting respectively the precursor mass and fragment mass tolerance to 20 ppm and 0.05 Da, the instrument to TOF and the peptide length from 8 to 50. Additionally, no cleavage enzyme was specified and the modifications (amidation of the peptide C-terminal, oxidation of methionine and pyro-glu formation from glutamine and glutamic acid) were defined as variable. Database searches against the UniProt reference proteome (UP000000589_10090) were performed in a similar manner. All searches (five brain samples from different mice in triplicate) were run on a Linux server.

The raw files were also analyzed with PEAKS Online (Bioinformatics Solutions Inc., Canada) with precursor tolerance set to 20 ppm and a fragment tolerance of 0.05 Da and no cleavage enzyme (unspecific digestion). Amidation, Deamidation (NQ), Oxidation (M), Pyro-glu from E and Q were set as variable modifications. Peptide to spectrum matches (PSMs) were filtered at 1% FDR. More search details can be found in Supplementary Table 1.

### Peptide Validation and Interpretation

In order to determine a reference list of identifications, the search results (mzid files) obtained with SearchGUI were analyzed with PeptideShaker (v2.0.27) (Vaudel et al., 2015) and the default PSM (Peptide-to-Spectrum Matches), Peptide and Protein Reports were extracted. Next to that, the same search results (mzid files) were used as input for the post-processing tool MS^2^ReScore (Silva et al., 2019) in a conda environment (Python 3.7) to predict the theoretical spectral intensities using the developers HCD model of the MS^2^PIP tool (Gabriels et al., 2019) for non-tryptic peptides with the fragment mass error set to 0.02 Da, expanding the number of features for further validation. Additionally, retention time predictions were added by the DeepLC tool (Bouwmeester et al., 2021) included in the MS^2^ReScore tool. As a final validation step, the different feature sets (search engine only (SE features), and search engine and MS^2^ReScore combined (All features)) were analyzed by Percolator (v3.05.0) (The et al., 2016) to improve the scoring between target and decoy sequences. All default parameters were applied at an FDR of 0.01.

Finally, the output files generated by MS^2^ReScore (Supplementary Data Sheets 1–5) were loaded into a Jupyter Notebook for further processing and interpretation. A summary table with all neuropeptide identifications (Supplementary Table 2) based on the MS^2^ReScore output was constructed as follows. First, the PSMs of the “All feature” set of each replicate individually were filtered for a q-value below 0.01. Secondly, the peptides identified by those filtered PSMs were grouped by identical peptide sequence (column of MS^2^ReScore output) and merged per brain section, resulting in a look-up table summarizing the MS^2^ReScore output on peptide level. This table was complemented with the information of the publicly available neuropeptide database Neuropep (Y. Wang et al., 2015) (downloaded at January 4, 2021^2^) to further filter and analyze the present neuropeptide identifications (Supplementary Table 2). A similar approach was performed to summarize the information for the sORF-encoded peptide identifications, where the grouped peptides files (as described above) were additionally grouped by protein Ids (column in output MS^2^ReScore) for each replicate individually before merging all replicates per brain type in a summary table. Furthermore, the identifications were supplemented with extra information extracted from the publicly available OpenProt database (version 1.5, based on protein Ids) and Ensembl (version 104, via BioMart based on gene names) repository and filtered for a protein length equal to or below 100 amino acids (aa) resulting in the final sORF-encoded peptide look-up table (Supplementary Table 3). A subset of non-coding identifications was generated out of the summary table by excluding all proteins identified on a gene of the type “protein-coding” according to added information. Of these identifications, the spectra were manually inspected in PeptideShaker for appropriate signal to noise ratio. Additionally, the coverage of the MS-peptide of the micropeptide was added and the PSM ids were checked for presence in the parallel searches against the reference proteome. Neuropeptide identifications and non-coding sORF selection was performed in a similar fashion as described above for the PEAKS search output, using the peptide reports filtered for a FDR below 0.01.

## Results

### Peptidomics Workflow With timsTOF

**FIGURE 1:**
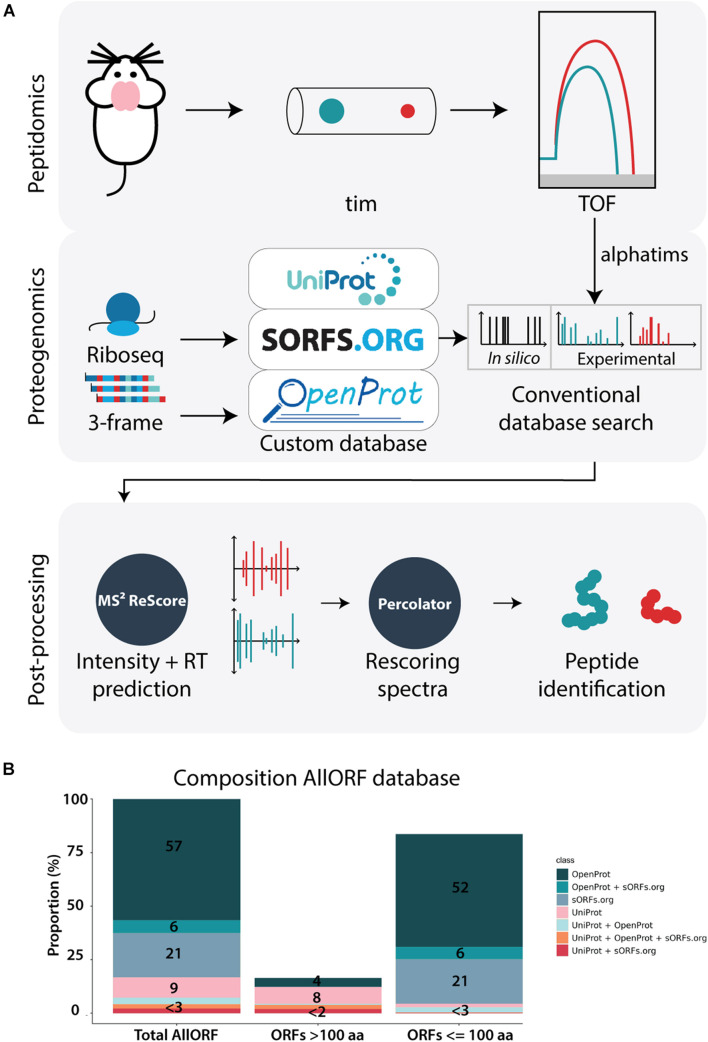
Peptidomics workflow with timsTOF. **(A)** The applied workflow consists of three major steps: 1. Peptidomics, where brain tissue samples are analyzed on the sensitive timsTOF MS, 2. Proteogenomics, where translatomics information from ribosome profiling data and 3-frame RNA translations is complemented with the reference proteome to create a custom search database for subsequently conventional database searching with MSGF+, and 3. Post-processing with machine-learning based tools. MS^2^ReScore adds spectral intensity and retention time predictions to the feature matrix later fetched into the post-processing tool Percolator, improving the scoring of target PSMs, leading to the list of peptide identifications. **(B)** The composition of the proteogenomics database used in this study. Out of the total 546,530 sequences present, 309,463 originate from OpenProt only, 32,106 overlap between OpenProt and sORFs.org and 113,446 exist only in sORFs.org. Next to that, 51,995 are unique to the reference proteome, while 16,430 overlap between the reference proteome and OpenProt, 11,744 between the reference proteome and sORFs.org and 11,346 were present in the three parts.

To explore the benefits of the trapped ion mobility Q-TOF MS-strategy in the context of neuropeptidomics, we devised a workflow integrating peptidomics, proteogenomics and machine-learning-based post-processing (Figure 1A). In the peptidomics analysis, peptides were carefully extracted from mouse brains and analyzed using a Bruker timsTOF Pro instrument. Then, experimentally generated spectra were matched to peptides using a conventional database search with a custom database constructed with ribosome profiling data and predicted three-frame RNA translations. The size of our custom database increased a 5-fold compared to the reference proteome search space. Out of the 546,530 sequences present, 83.3% (455,015) originate from a proteogenomics source, while only 9.5% (51,995) are derived from the annotated proteome and 7.2% (39,520) from the annotated proteome overlapping with predicted proteins. Additionally, the majority (83.6%) consists of proteins smaller than 100 AA, with over 50% originating from OpenProt predictions and 20% based on ribosome profiling predictions of sORFs.org (Figure 1B). In the last step of the workflow, several machine learning-based tools were applied to further increase the peptide identifications. More specifically, the feature set generated for each PSM by the search engine MSGF+ during the database searching was expanded with additional features obtained from MS^2^ReScore. Next to the fragment ion peak intensities predicted by MS^2^PIP, retention times predicted by DeepLC were included, finally increasing the information for every PSM from 26 to 103 features. This information matrix was subsequently fetched into the semi-supervised post-processing tool Percolator, that used this data to learn a new scoring function to accurately separate decoy from target PSMs as a final step of the workflow.

### Feature Predictions in a Peptidomics Context

**FIGURE 2:**
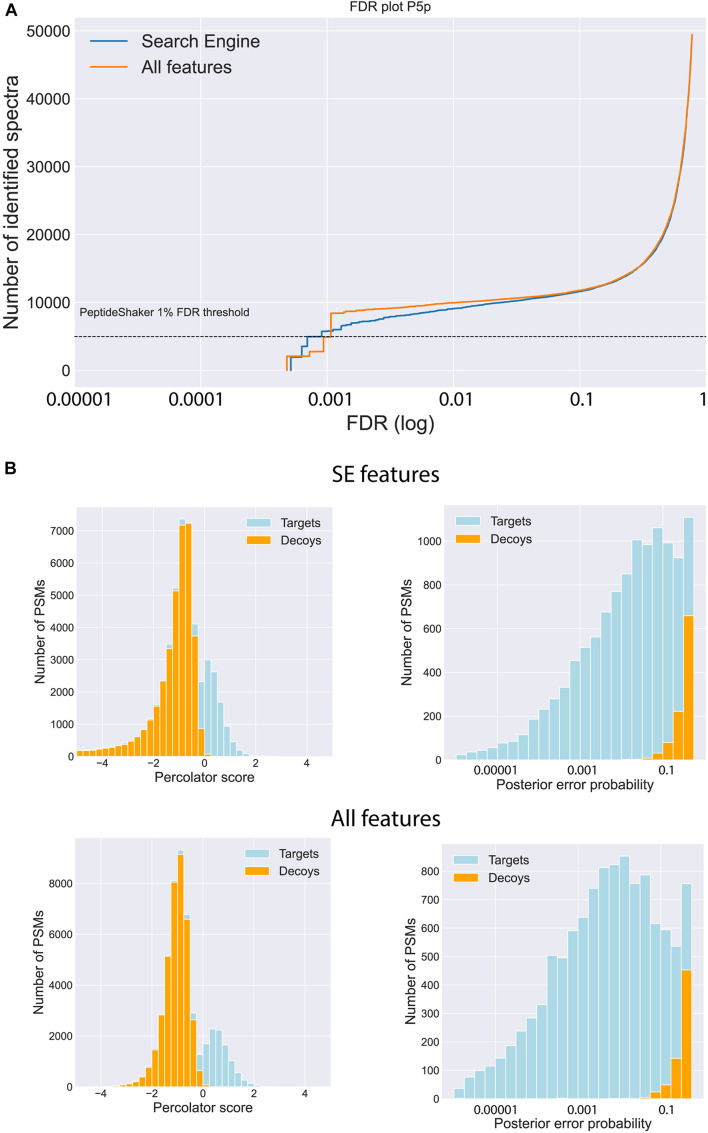
Impact of feature prediction on PSM level. **(A)** Visualization of the number of identified spectra for a range of FDRs when using only the features generated by MSGF+ (Search Engine, blue) in Percolator and using the features of MSGF+ and the predicted features generated by MS^2^PIP and DeepLC (All features, orange) in Percolator. **(B)** Increased discrepancy power between targets and decoys is illustrated by an increase of the Percolator score of the target PSMs and decrease of the posterior error probability (PEP) when including all the features (bottom row) compared to solely the search engine features (upper row). (Data of posterior brain samples is shown, similar graphs for the other four brain samples are found in Suppl. Fig. 1-4).

The effect of the post-processing tools and the expanded feature set on the final identifications was evaluated on several layers, starting with the PSM level. The incorporation of the Percolator tool led to a considerable increase of PSMs with a false discovery rate (FDR) below 0.01 at PSM level (overview in Supplementary Table 1). When using the search engine (SE) feature set in combination with Percolator, the number of valid PSMs below the PSM FDR threshold of 0.01 is almost doubled compared to the number of identified PSMs obtained with only PeptideShaker (PS), lacking the rescoring power of Percolator (Figure 2A and Supplementary Figures 4–7 and Table 1). The addition of the predicted spectral intensities and retention times to the feature set, further referred to as “All features,” only marginally impacted the total number of identified PSMs (2% more PSMs on average). Despite the limited increase of validated PSMs, the gain of additional information is evidenced by a higher confidence in the identifications, visualized by the increased Percolator scores and decreased posterior error probabilities (PEP) for the target PSMs (Figure 2B and Supplementary Figures 4–7). Additionally, MS^2^PIP and DeepLC features are among the top 10 features contributing to the Percolator rescoring function (Supplementary Figures 1–3).

**TABLE 1:** Overview general sORF identifications identified by the MS^2^ReScore approach.

Category	sORFs	Total PSMs	Total pept	Non-coding	Total PSMs	Total pept
SWISS	24	1,235	516	2	2	2
TrEMBL	16	1,989	562	2	6	8
OpenProt predicted	105	128	123	44	52	46
sORFs.org predicted	22	45	41	0	0	0
Total sORFs	167	3,397	1,242	48	60	56

**FIGURE 3:**
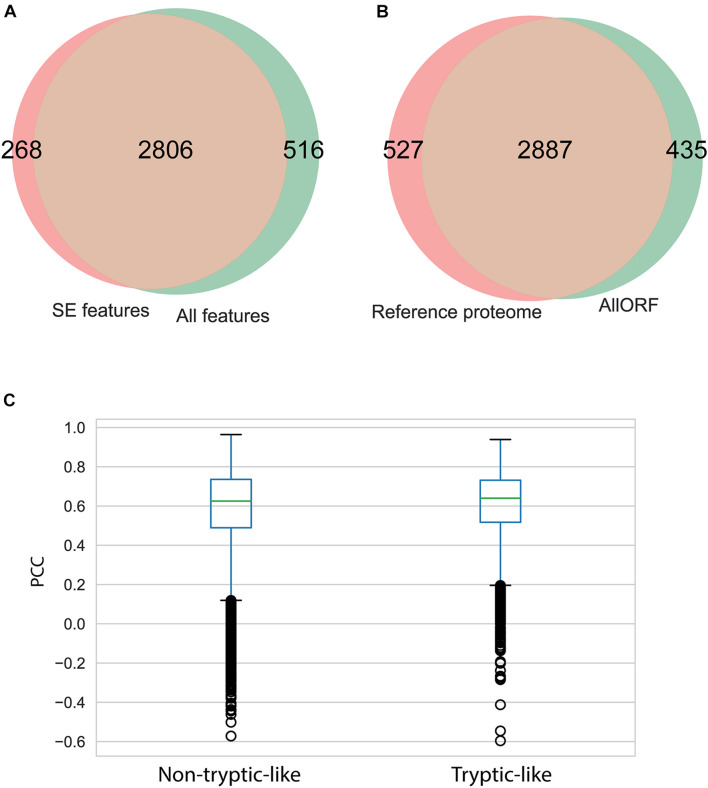
Impact of feature prediction on peptide level. **(A)** Overlap of unique peptides identified considering “All features” and considering only the “SE features” during proteogenomic searches. **(B)** Overlap of unique peptides identified searching against the custom proteogenomics database versus the functional proteome (Uniprot). **(C)** Boxplot of the Pearson correlation coefficient (PCC) of the predicted spectrum intensities by MS^2^PIP and the observed spectrum intensities, for the tryptic peptides (ending on R or K) and non-tryptic-like peptides (others).

The influence of the “All features” set is further noted by a slight increase of the peptide identifications. In total, 3,322 unique peptides were identified across the five brain sections using the “All features” set. Of those, 84% overlapped with the 3,074 unique peptides identified with only the SE features and Percolator. Both sets resulted in a small fraction of peptides solely identified with respective features (Figure 3A). To investigate the impact of the database size, the MS-identified peptides were compared to the peptides identified during a database search with only the UniProt Mouse reference proteome as search space. The proteogenomic search resulted in a similar number of peptides identified (Figure 3B) with the reference searches. Next to using an enlarged search space, non-tryptic peptides form an additional challenge during peptidomics studies. To evaluate the performance of the intensity predictions by MS^2^PIP (Supplementary Figures 8, 9), the Pearson correlation coefficients (PCC) were investigated for both tryptic-like (basic aa at the C-terminus) and non-tryptic-like peptides. This coefficient is calculated by comparing the predicted intensities to the corresponding empirical spectra. Here, around 80% of the identified peptides are considered non-tryptic-like (not ending on R or K). These peptides resulted in lower Pearson correlations in contrast to the tryptic-like peptides, but only slightly so (Figure 3C).

### Neuropeptides and sORF-Encoded Peptides

**FIGURE 4:**
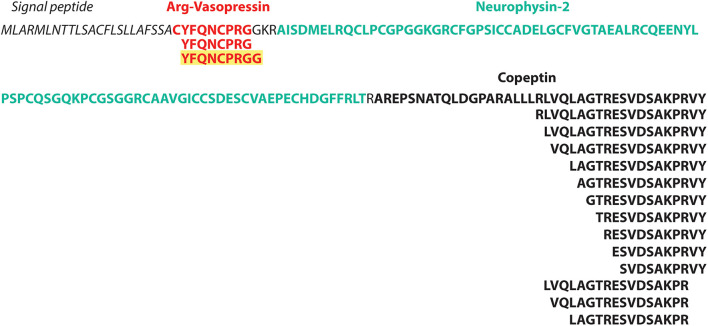
timsTOF data enables study of endogenous proteolytic processes. Illustration of the neuropeptide precursor Vasopressin-neurophysin 2-copeptin (P35455) and the annotated peptides as reported in Uniprot in bold. Below are the MS-identified peptides illustrating the proteolytic process. The peptide in yellow was identified in this study, but not present in the Neuropep reference database or annotated in Uniprot.

In total, 346 unique MS-peptides supported by 4,645 valid PSMs were identified from 31 known neuropeptide precursors. Cross-referencing the sequences of the identified peptides with the publicly available neuropeptide database Neuropep (Wang et al., 2015) revealed 66 different known neuropeptides from 22 different families. Members of the highly expressed secretogranins, proSAAS and cholecystokinin families were detected in addition to cerebellins, galanins, Neuropeptide Y and vasopressins (Supplementary Table 2). Of those peptides, 20 peptides were identified on five different neuropeptide precursors (Penk, Vip, Scg2, Nmb, and POMC) but without sequence overlap with the refence sequences present in the Neuropep database. For those, the name of the annotated neuropeptide was added based on UniProt annotations. Lastly, an additional 55 peptides were identified on the Neurosecretory protein VGF (Uniport accession Q0VGU4). This precursor was not included in the Neuropep database for mouse but is well documented for *Rattus norvegicus* and *Homo Sapiens*. All together, we identified a total number of 401 peptides originating from known neuropeptide precursors with our described method (Supplementary Table 2). However, PEAKS analyses on peptide level resulted in 805 PSMs and 153 peptides extra (Supplementary Table 2). Despite these higher number of identifications, only one family of neuropeptides, Sauvagine/corticotropin-releasing factor/urotensin I, was missed by the MS^2^ReScore method, while two other families, Galanins and Nucleobindins, were lacking in the list of peptides identified with PEAKS. In total, 344 peptides including different modifications were identified by both methods, while 203 and 60 were unique to PEAKS and MS^2^ReScore respectively. Additionally, both methods succeeded in capturing several truncated peptides only single amino acids different in length, illustrating the endogenous proteolytic processing (Figure 4). Next to the peptides originating from known neuropeptide precursors, our proteogenomics database was designed to enable identification of new sORF-encoded peptides. We obtained 1,277 unique protein ids throughout all experiments, of which 167 were proteins with a length below or equal to 100 aa. Of these, 40 were already annotated in Uniprot or TrEMBL, while 127 were predicted sORFs from OpenProt or sORFs.org (Table 1). These sORFs were identified with a total of 1242 peptides originating from 3397 PSMs. As a comparison, the peptide reports of PEAKs lead to 213 ids with a length below or equal to 100 aa, of which 132 were predicted by OpenProt or sORFs.org. Since we take particular interest in sORFs predicted in non-coding regions, out-of-frame sORF predictions on fully characterized known protein-coding genes were excluded for further analysis. Of the OpenProt predictions, 44 of these sORFs were located on transcripts assigned as either long-non-coding RNA (lncRNA) (17), To be Experimentally Confirmed (TEC) transcripts (9) and pseudogenes (14). Four additional sORFs were considered in the selection since they are located on predicted genes (Gm1141, Gm14391, 4933416I08Rik, 2900026A02Rik) with limited experimental evidence. In addition, two identifications in the Uniprot group, the protein product on the RIKEN cDNA 1500009L16 gene [overexpressed in colon carcinoma 1 protein homolog (OCC-1)] and the UPF0729 protein C18orf32 homolog on the BC031181 gene, were also included for further analysis due to the limited public information on either gene. Finally, two uncharacterized proteins (Q3TP86 and Q9CU37) in UniProt completed our short list of 48 sORFs identified in a non-coding context by the MS^2^ReScore approach (Table 1 and Supplementary Table 3). A total of 60 PSMs and 56 peptides (Supplementary Table 3) resulted in the MS identification of these sORFs. Only five sORF-encoded peptides were identified with more than one PSM. To avoid erroneous identifications, additional information was added to increase the confidence of the identifications serving as validation steps. First, the MS-identified sequence tags were tested for uniqueness by the Blastp algorithm against the *Mus Musculus* consensus database. Four sequence tags, two from the Swiss-TrEMBL group and two predicted sORFs, resulted in hits with an e-value below 10^–10^, further confirming the uniqueness of the other sequence tags. Secondly, all MS-spectra were manually evaluated and for every PSM id, the occurrence in the parallel reference proteome searches was verified. None of the PSMs providing MS evidence for a presumable non-coding sORF-encoded peptide was assigned during the searches against the reference proteome. Additionally, 17 of the candidates were covered for more than 30% by the identified MS-peptide, while only 2 were only covered for less than 10% (Supplementary Table 3). Finally, the 48 non-coding sORFs were inspected for extra indications supporting their biological relevance. A Blastp search against all proteomes revealed significant hits with an e-value below 10^–10^ for 17 sORFs, hypothesizing conservation for those and thus biological relevance. For 10 of the predicted sORFs and the two sORFs of the Swiss category, the OpenProt repository reported homology in other species and for one of them, IP_950537, previous MS evidence was also reported. We further inspected the presence of specific biochemical properties such as short disordered regions and transmembrane helices (features sometimes described to be enriched in sORFs). From these predictions, seven sORFs contain a transmembrane region, while all but two possessed some evidence of short disordered regions according to IUPred2A (Table 2). Taken together, our strategy resulted in MS-evidence for 127 predicted sORFs of which 48 sORFs on non-coding regions and additional features hinting toward their translation. Next to our method, 40 sORFs on presumably non-coding locations were identified based on the analysis with the PEAKs software (Supplementary Table 3). Of those, only four were detected by both methods, namely three identifications for the Uniprot group (OCC-1, UPF0729 protein C18orf32 homolog and uncharacterized protein Q9CU37) and only one predicted sORF (IP_871601) situated on the predicted Gm19033 gene. Two additional uncharacterized proteins from the UniProt group (Q3V047 and A0A5F8MQ94), located on predicted gene Gm10640 and the lncRNA 1810058I24Rik respectively, were supported by MS-peptides identified with PEAKS, as well as 34 other sORFs predicted by OpenProt, located on predicted protein coding genes (1), lncRNAs (7), TECs (4), pseudogenes (20), and other non-coding RNA types (2). Although PEAKS supported four sORFs of the MS^2^ReScore selection with additional information, 21 sORFs uniquely found in the PEAKS analysis are solely identified by a single peptide and a single PSM, illustrating the challenges in accurately identifying sORFs on non-coding regions. In conclusion, analysis of our timsTOF data from the mouse brain by two independent strategies resulted in MS-evidence for 84 potential sORFs on presumed non-coding locations and offers a pool of potential biological candidates for further studies.

**TABLE 2:** Additional features for sORFs identified from non-coding regions by MS^2^ReScore and PEAKS.

Category	Total	BLASTp	MS evidence	TE evidence	Orthology	TMH
Swiss	2	2	2	2	2	0
TrEMBL	4	4	No info	No info	No info	0
OpenProt	78	31	1	5	22	9
Total	84	37	3	7	24	9

*A summary of the number of sORFs on non-coding regions with additional features hinting toward their biological relevance. MS evidence, TE (translation efficiency) evidence and Orthology are extracted from the OpenProt repository, while TMH (transmembrane helix) was predicted by the online tool TMHMM Server v. 2.0.*

## Discussion

In this explorative study, we integrated novel developments in the peptidomics and proteogenomics fields to study the neuropeptidome of mice. Next to the total of 607 peptides originating from known neuropeptide precursors, many of which performing fundamental biological roles (Le et al., 2013; Hayakawa et al., 2019), we were interested in identifying sORF-encoded peptides from so-called non-coding regions. Over the last years, the number of sORFs predicted by conservation tools (Mackowiak et al., 2015) or ribosome profiling data (Erhard et al., 2018; Olexiouk et al., 2018; Brunet et al., 2021) has increased significantly, while the number of functionally characterized sORF-encoded peptides still remains rather limited. One potential explanation is that it is challenging to confidently identify these sORF-encoded peptides using proteomics techniques. Much efforts have focused on optimizing MS-based workflows (Bartel et al., 2020; Peng et al., 2020; Fabre et al., 2021), where a traditional bottom-up strategy with an enzymatic digestion prior to MS-analysis has often been favored (Fabre et al., 2021). Several adaptations of the separation (Kaulich et al., 2020) or enrichment (Petruschke et al., 2020) and digestion methods (Kaulich et al., 2021) have been investigated and further applied in a wide variety of biological settings, but only a limited number of studies consider the top-down strategy as a potential workflow in SEP discovery (Li et al., 2017; Budamgunta et al., 2018; Cassidy et al., 2021). Nevertheless, unlike bottom-up workflows, this alternative method delivers valuable information about proteoforms, C- or N-terminal specific characteristics and post-translational modifications (Cassidy et al., 2021) that are linked to biological functions. Additionally, the potential limited number of tryptic peptides present in SEPs (Kaulich et al., 2021) or short neuropeptides is counteracted by omitting the enzymatic digestion prior to analysis. Here, 48 sORFs of 84 sORFs identified on non-coding regions are peptides without a basic C-terminus, which would have been lost during conventional bottom-up strategies. Next to SEP identification, top-down strategies were originally introduced to study peptide hormones and neuropeptides (Romanova and Sweedler, 2015). The proteolytic processing of precursors proteins into these peptides by specific proteases is fundamental for their bioactive function, but pinpointing the cleavage sites and the peptidases involved remains challenging with bottom-up workflows. Since top down MS approaches omit pre-processing with a digestion enzyme, it opens up opportunities to study naturally occurring degradation products (Kim et al., 2012). In this study, we illustrated the potential of timsTOF data to further facilitate this research. Besides the detection of fragments of neuropeptides demonstrating the proteolytic process (Figure 4 and Supplementary Table 2), we managed to detect new sequences not present in the widely used reference Neuropep (Wang et al., 2015).

Another key element in SEP discovery is the efficient separation of proteins to reduce the sample complexity and thus increase the SEP sensitivity (Petruschke et al., 2020; Cassidy et al., 2021). In this study, we employ the relatively recent timsTOF technique (Lubeck et al., 2018), where an increased sensitivity is achieved by separation, trapping and accumulation of peptide ions based on ion mobility (Lubeck et al., 2018). The mobility of an ion is determined by the three-dimensional shape and charge in the gas phase, adding an additional separation dimension extra to mass over charge ratio and retention time (Meier et al., 2018). Also, the combination of trapped ion mobility spectrometry (tims) and Parallel Accumulation Serial Fragmentation (PASEF) effectively allows to maximize both throughput and sensitivity. This new technology has recently been used for analysis of complex samples in different research fields, such as the exploration of the proteome in human cerebrospinal fluid (Macron et al., 2020), detection of antibacterial peptides in the human endometrial fluid (Azkargorta et al., 2020) or the proteome of the malaria parasite *Plasmodium* (Hamada et al., 2021). To our knowledge, this is the first study where timsTOF is applied for neuropeptide and sORF discovery and only the second top-down MS study where non-digested peptide samples are identified using a timsTOF. The other study using top-down sample developed a new method for antibody-drug conjugate identification (Larson et al., 2021). To accommodate the four-dimensional data space (4D) of timsTOF data, mass over charge, retention time, intensity and ion mobility, specific modifications were newly implemented to commonly used search engines. The search engine MSFragger was adapted to process timsTOF data and supplemented with a new quantification tool IonQuant (Yu et al., 2020), while a new algorithm was implemented alongside the commonly used MaxQuant software to extract 4D features also enabling a new matching between runs algorithm (Prianichnikov et al., 2020). A third option for timsTOF analysis is the *de novo*-based PEAKS software. In the current workflow, we used the robust search engine MSGF+ due to its advantages in peptidomics searches (Kim and Pevzner, 2014; Sherafat et al., 2020) and compatibility with our post-processing tools of choice (Silva et al., 2019). But the integration of more specialized search engines that omit data manipulation prior to searches like MaxQuant or MSFragger into the MS^2^ReScore pipeline in the future could further expand the information extracted from timsTOF data and advance neuropeptide and sORF-encoded peptide identification.

One of the major drawbacks of peptidomics and proteogenomics is the enlarged search space, leading to reduced sensitivity and increased false positive rates (Maes et al., 2019; Verbruggen et al., 2021). To overcome this drawback, Hayakawa et al. (2019) reduced the search space based on predicted neuropeptides enabling a more sensitive peptide identification, but this approach is limited to samples purified from larger proteins and less sensitive MS data. Another recent proteomics study (Verbruggen et al., 2021) used a search space of up to 50 times the size of their reference proteome, but achieved accurate and confident identifications by applying machine-learning based rescoring tools. In our study, we applied a similar strategy with no increased identification using a reduced search space (Figure 2B). Additionally, we observed an improved separation between target and decoy spectra, shown by increased Percolator scores and lower PEP (Figure 2). While the application of machine-learning based tools partly compensated for the enormous search space, we here found that, unlike previously reported (Verbruggen et al., 2021), the integration of spectral intensity and retention time prediction are only responsible for a marginal increase of valid PSMs. We hypothesize that this discrepancy can be explained by the different nature of the spectra used to train the spectral intensity prediction model of MS^2^PIP and the spectra generated by timsTOF MS or the difference in complexity between proteomics and peptidomics samples. An evaluation of three available MS^2^PIP models trained for different fragmentation methods (HCD, TOF, and CID) fails to pinpoint an outperforming model for our timsTOF data (Supplementary Table 8). Additionally, another factor influencing fragmentation patterns is the length and charge of the peptides (Huang et al., 2008, 2005; Ramachandran and Thomas, 2020). Since no digestion enzyme is used, peptidomics harbor longer and more highly charged peptides leading to an extra challenge for spectral intensity predictions. A recent comprehensive comparison of several available spectral intensity prediction tools illustrates the decrease of prediction accuracy for the machine learning-based tool MS^2^PIP compared to deep learning methods as the peptide length and charge increases (Xu et al., 2020). These conclusions were drawn based on analyses with tryptic shotgun proteomics, but we hypothesize that these effects are even more prominent in a peptidomics context. Indeed, the non-tryptic model used in this study performs slightly better for longer and highly charged peptides (Supplementary Figure 9). A recent attempt was made to improve predictions for double and triply charged tryptic peptides by using a deep convolutional neural network (Lin et al., 2019) but a general model for peptidomics data is currently lacking. Thus, in order to fully benefit from the additional information that spectral intensity predictions can provide, a new model should be trained tailored to timsTOF data in a peptidomics context. This can only be done when sufficient timsTOF datasets become available in the future. Together with a recent study to identify antibody-based drug products (Larson et al., 2021), this is only the second study where top-down strategies are analyzed on a timsTOF pro mass spectrometer. Alternatively, the spectral intensities could be predicted by a different algorithm, that is reported to be more robust toward different data characteristics, like PROSIT (Gessulat et al., 2019). This deep neural network was originally trained on tryptic peptides, but succeeds in predicting spectra from non-tryptic peptides, as well as spectra from data-independent acquisition settings (Xu et al., 2020). Although both prediction tools performed equally well in a proteogenomics study (Verbruggen et al., 2021), the flexibility of PROSIT could be an advantage to analyze more challenging timsTOF and peptidomics data. As the machine learning field continues to evolve and more tools become available, more timsTOF-specific properties could be predicted and incorporated as additional information next to the spectral intensity and retention time described in this study. For example, a newly published model (Meier et al., 2021), based on a deep recurrent neural network trained with timsTOF data, can now predict the collisional cross section (CCS) values for any peptide. Since these values can be derived from the ion mobility, this feature is a promising characteristic to include. More so, the CCS values are largely independent of experimental circumstances, so they are highly precise and reproducible.

In this study, we aimed to identify sORF-encoded peptides, and more specifically on non-coding regions. We chose to strictly apply the “non-coding” filter on gene level instead of the more commonly used transcript level to avoid identifications supported by experimental evidence originating from overlapping protein-coding regions mistakenly considered as non-coding hits. With this stringent setting, 84 potential sORF-encoded peptides on non-coding regions or regions with limited information were detected with MS evidence. Several of these contain additional features supporting their translation and potential biological importance (Table 2), such as disordered regions, that are reported to be enriched in SEPs (Mackowiak et al., 2015) and transmembrane helices, which are essential in a wide variety of processes like cell-cell communication (Makarewich, 2020). Besides the technological challenges during SEP discovery, the in-depth validation of spectra and identified peptides remains a hurdle that needs to be overcome. Several attempts, both in bottom-up (Slavoff et al., 2013) and top-down approaches (Budamgunta et al., 2018) are made to construct a robust method for validation of so called “one-hit wonders,” identifications based on only one PSM. As discussed above, the properties of timsTOF fragmentation spectra in a peptidomics context might differ from other MS-methods, indicating the need for adjusted validation criteria for this specific data. One potential solution to validate spectra from one-hit wonders is the comparison between the experimental spectra of the endogenous peptide and the spectra from its chemically synthesized counterpart (Chandra et al., 2020). This in combination with parallel reaction monitoring (PRM) successfully validated one-hit wonder missing proteins in the human spermatozoa proteome (Vandenbrouck et al., 2016). In our study, we manually inspected the spectra, together with some additional validation steps and managed to identify several previously described sORF-encoded peptides (D’Lima et al., 2017; Budamgunta et al., 2018; Bhatta et al., 2021). Among those was the mouse equivalent of the human micropeptide Nobody which interacts with the mRNA decapping complex (D’Lima et al., 2017). It was identified on what was previously thought to be a lncRNA transcript. Likewise, another sORF-encoded peptide identified in our study, the mitochondrial transmembrane micropeptide Mm47 was localized on lncRNA 1810058I24Rik (Bhatta et al., 2021). This recently characterized micropeptide is required for the activation of the Nlrp3 inflammasome, indicating a vital biological function. This illustrates the growing number of SEPs on locations previously thought to be non-coding included into the reference proteome due to intense research over the past years. This, together with our strict filter for the non-coding gene type and the incomplete analysis of timsTOF data with the available prediction tools, leads to a modest number of SEPs identified hereof potential SEPs identified here that require further validation to exclude false positive hits. Potential other MS-based validation strategies to limit false positive hits and thus increase the confidence of SEPs on non-coding regions are selected reaction monitoring (SRM) and PRM for individuals SEPs, while the biological mechanism could be studied by the identifications of interaction partners (J. Chen et al., 2020) with several techniques (reviewed in Peeters and Menschaert, 2020) or with the analysis of knock-out or knock downs of the specific genes. For example, a CRISPR-based screening strategy revealed an essential biological function for hundreds of non-canonical coding sequences in human cells (Chen et al., 2020). Besides knock-out or knock downs of the precursor protein to evaluate biological effect, the bioactivity of neuropeptides can be assessed by specific assays (reviewed in Corbière et al., 2019). A recent study (Palkeeva et al., 2019) generated structural analogues of Galanin peptides to investigate the biological activity of new forms in comparison to previously described ones. Among the tested sequences was one detected in this study, solely by the MS^2^ReScore approach. By using rat model of myocardial I/R injury *ex* and *in vivo*, the peptide was reported to exert cardioprotective properties. All together, these identifications demonstrate the robustness of our method and the potential biological functions that sORF-encoded peptides originating from non-coding transcripts can exercise.

In conclusion, we managed to overcome some of the well-known challenges in peptidomics and proteogenomics studies by integrating machine-learning based tools into our post-processing workflow leading to the identification of a wide set of neuropeptides and sORF-encoded peptides with a focus on the ones in a non-coding context. However, the full potential of the sensitive timsTOF Pro MS will further benefit from specialized timsTOF prediction models for spectral intensities combining timsTOF specific features such as CCS. A tailored validation strategy is also recommended to exclude false positives as well as in-depth follow-up analysis to explore the biological function. Combining these adaptations in future workflows will lead to a better coverage of the neuropeptidome and sORF-encoded peptides.

## Data Availability Statement

The raw data and processed datasets presented in this study can be found on ProteomeXchange with accession number PDX026584 (http://www.proteomexchange.org/accession:PDX026584).

## Ethics Statement

The animal study was reviewed and approved by ECD University of Antwerp (UA ECD2019-50).

## Author Contributions

GM and GB designed and supervised the research. MP and KB analyzed the data and wrote the manuscript. RG helped in applying MS^2^ReScore and provided feedback on the obtained results. EP collected and prepared the samples and ran the MS experiments. All authors contributed to the article and approved the submitted version.

## Conflict of Interest

GM is a co-founder of OHMX.bio, Ghent, Belgium. The remaining authors declare that the research was conducted in the absence of any commercial or financial relationships that could be construed as a potential conflict of interest.

## Publisher’s Note

All claims expressed in this article are solely those of the authors and do not necessarily represent those of their affiliated organizations, or those of the publisher, the editors and the reviewers. Any product that may be evaluated in this article, or claim that may be made by its manufacturer, is not guaranteed or endorsed by the publisher.

## Footnotes

1 GitHub - MannLabs/alphatims: An open-source Python package for efficient accession and analysis of Bruker TimsTOF raw data from the Mann Labs at the Max Planck Institute of Biochemistry.

2 Neuropeptide Database - Browse (isyslab.info).

## Supplementary Material

Supplementary Figure 1 Weights of Percolator Rescoring model. Plot of the features (only with absolute value >1) used in the Percolator model to rescore PSMs for the posterior brain sample (replicate A).

Supplementary Figure 2 Idem as 1 (replicate B).

Supplementary Figure 3 Idem as 1 (replicate C).

Supplementary Figure 4 Impact of feature prediction on PSM level for total brain section.

Supplementary Figure 5 Impact of feature prediction on PSM level for anterior brain section.

Supplementary Figure 6 Impact of feature prediction on PSM level for cerebellum brain section.

Supplementary Figure 7 Impact of feature prediction on PSM level for cortex brain section.

Supplementary Figure 8 Evaluation of the available MS^2^PIP models of the Pearson Correlation Coefficient for four different models, trained with tryptic peptides of different fragmentations (CID, HCD, TOF) and the non-tryptic model used in this study for b and y-ions combined **(A)**, only y-ions **(B)** and only b-ions **(C)**.

Supplementary Figure 9 Evaluation of peptide of lengths and charge. Boxplots of the Pearson Correlation Coefficient for the different MS^2^PIP models.

Supplementary Table 1 Summary Search Results.

Supplementary Table 2 Neuropeptides identifications.

Supplementary Table 3 sORFs identifications.

Supplementary Data Sheet 1–5 MS^2^ReScore output.
