# Supplementary figures and images for: Ion Mobility Coupled to a Time-of-Flight Mass Analyzer Combined With Fragment Intensity Predictions Improves Identification of Classical Bioactive Peptides and Small Open Reading Frame-Encoded Peptides
Source: Front Cell Dev Biol. 2021 Sep 17;9:720570. doi: 10.3389/fcell.2021.720570 (PMC8484717; doi:10.3389/fcell.2021.720570)

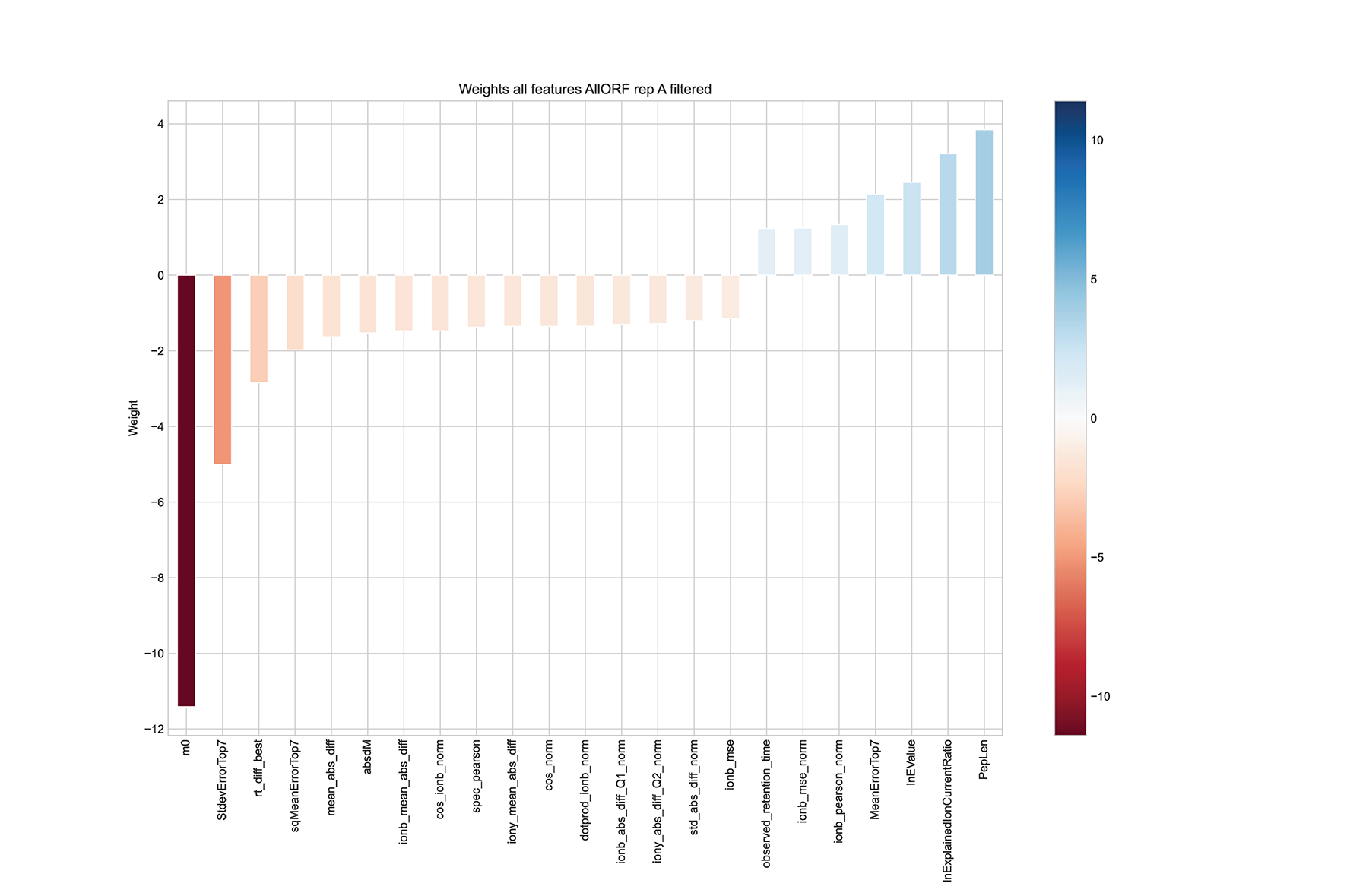

Supplement: Supplementary Figure 1 — Weights of Percolator Rescoring model. Plot of the features (only with absolute value >1) used in the Percolator model to rescore PSMs for the posterior brain sample (replicate A). [file Image_1.TIF]

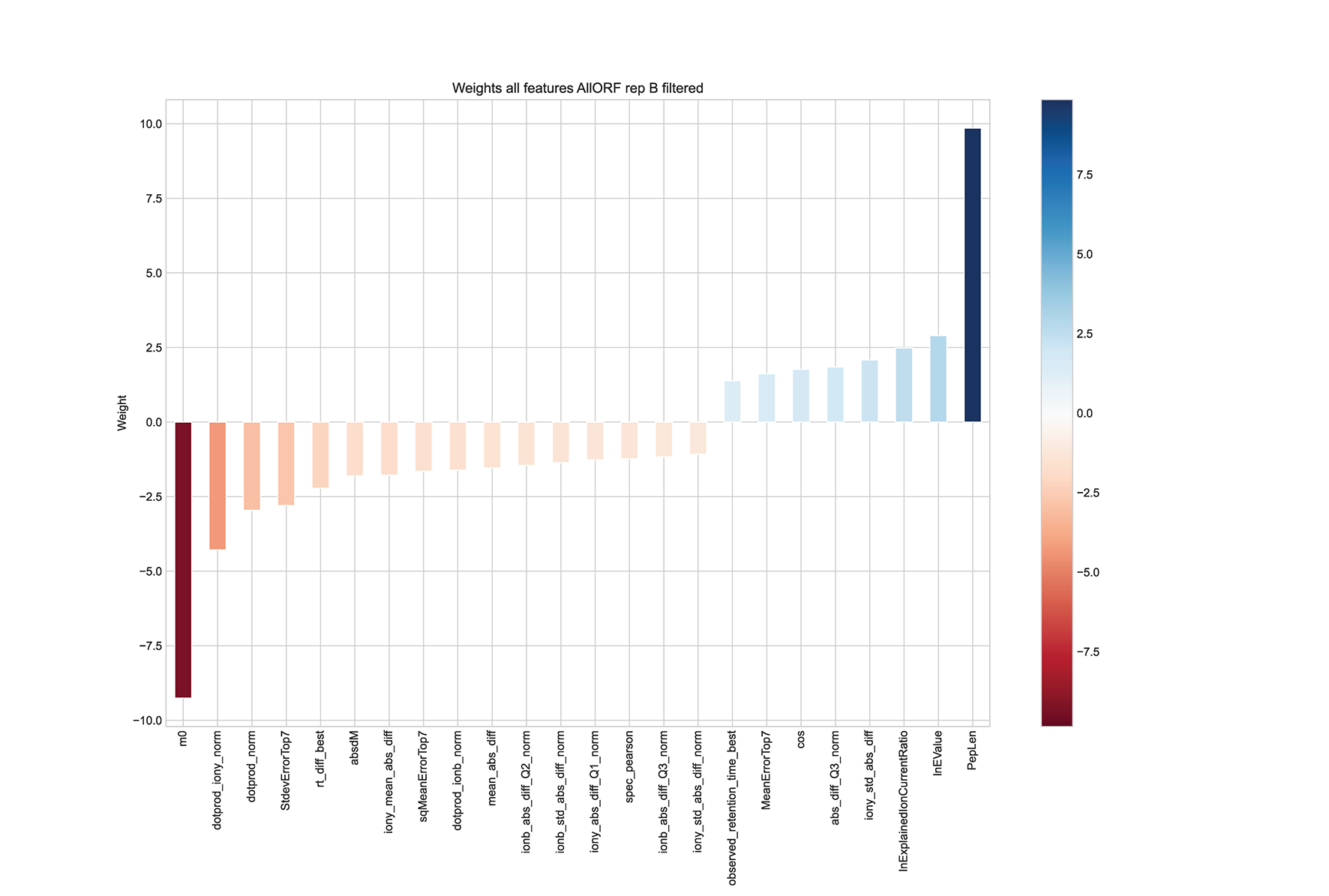

Supplement: Supplementary Figure 2 — Idem as 1 (replicate B). [file Image_2.TIF]

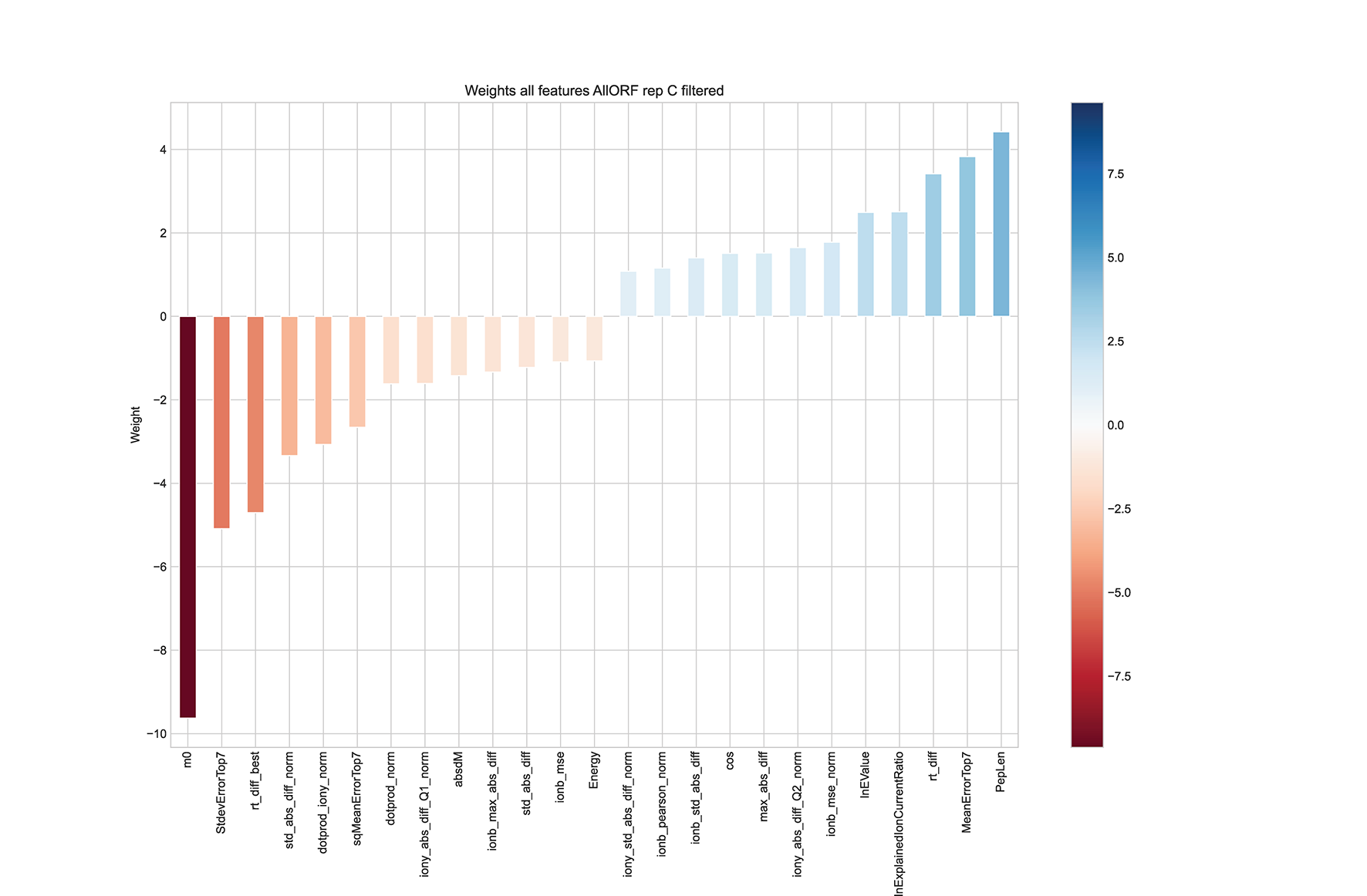

Supplement: Supplementary Figure 3 — Idem as 1 (replicate C). [file Image_3.TIF]

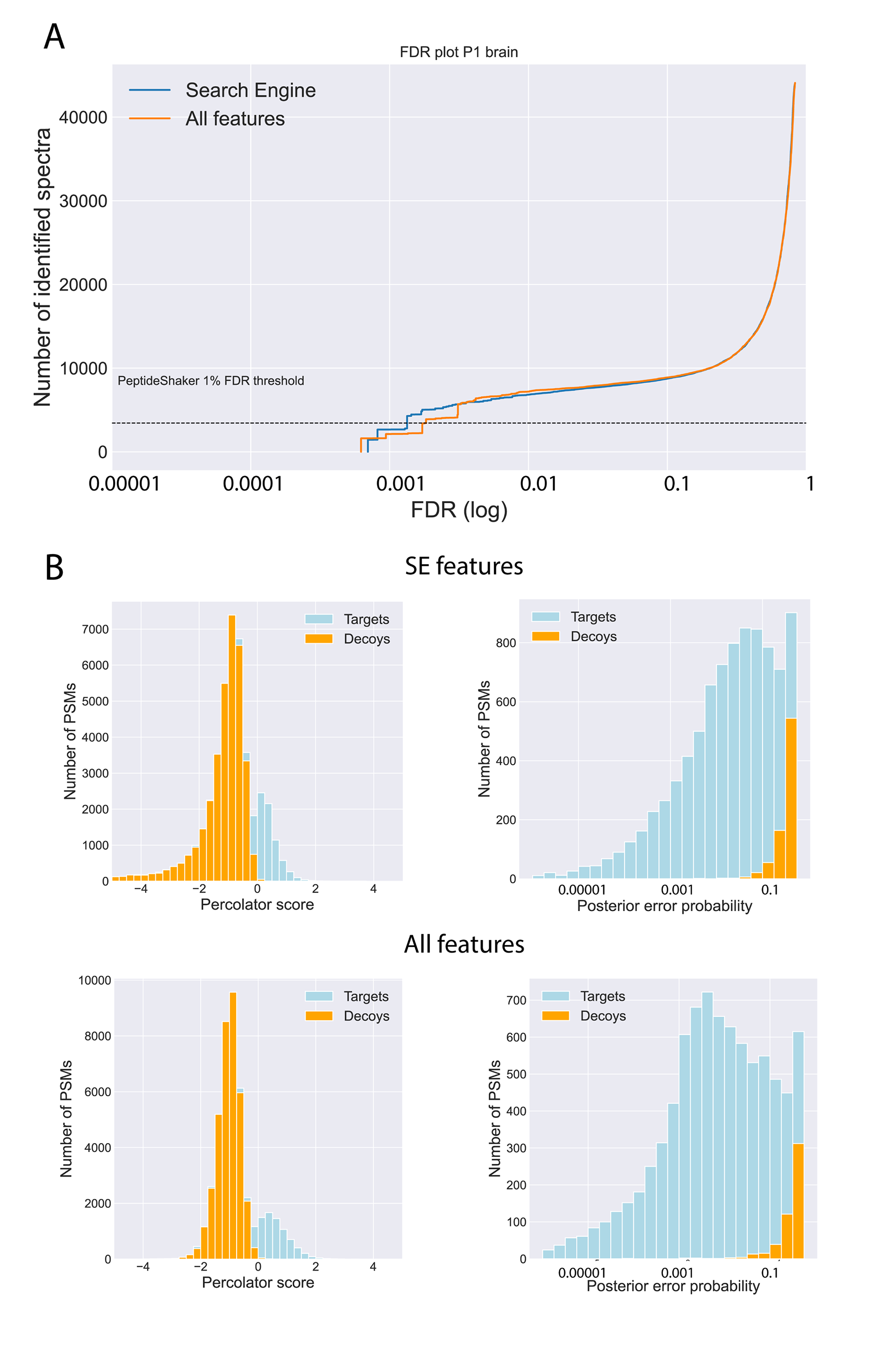

Supplement: Supplementary Figure 4 — Impact of feature prediction on PSM level for total brain section. [file Image_4.TIF]

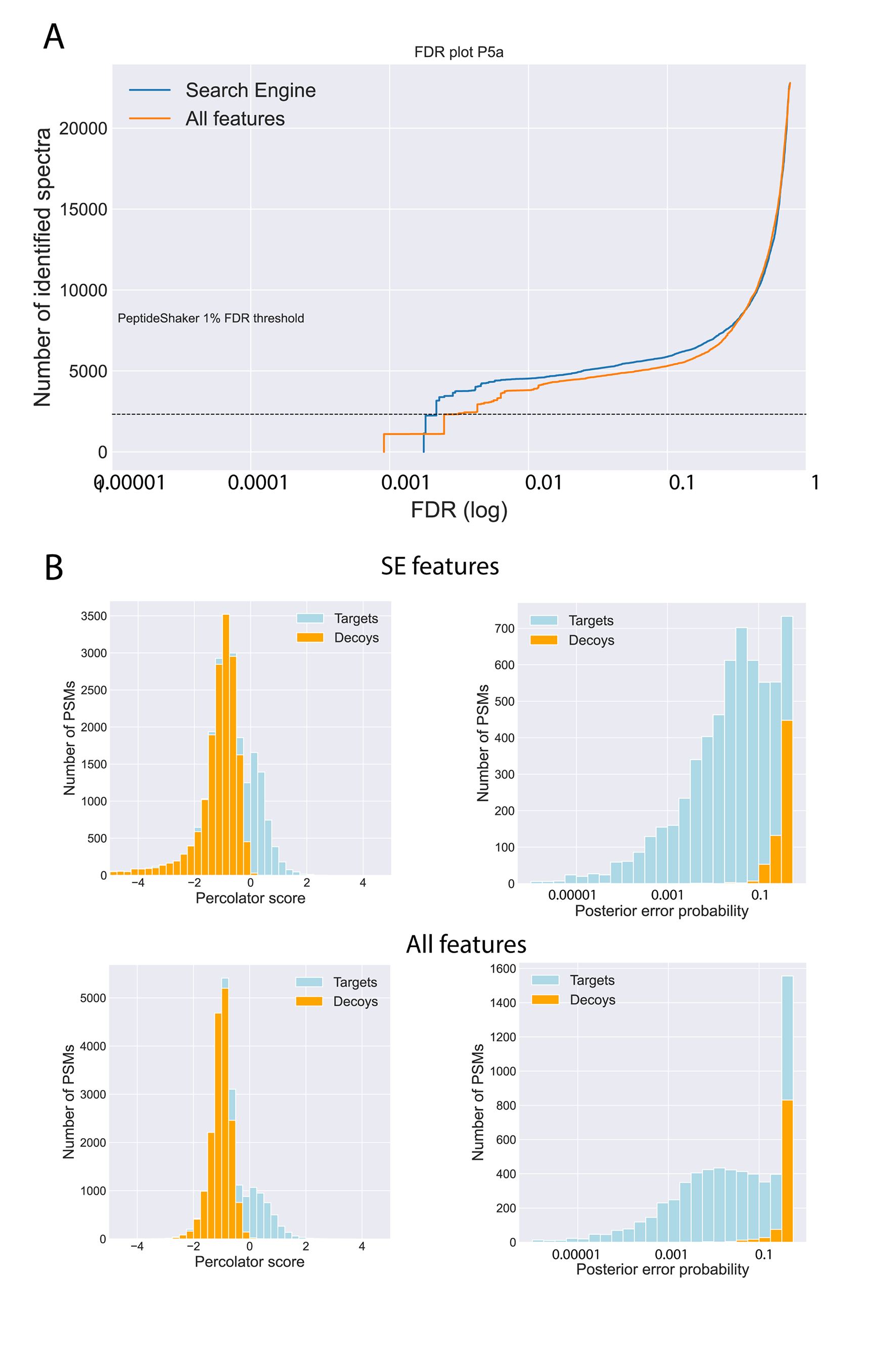

Supplement: Supplementary Figure 5 — Impact of feature prediction on PSM level for anterior brain section. [file Image_5.TIF]

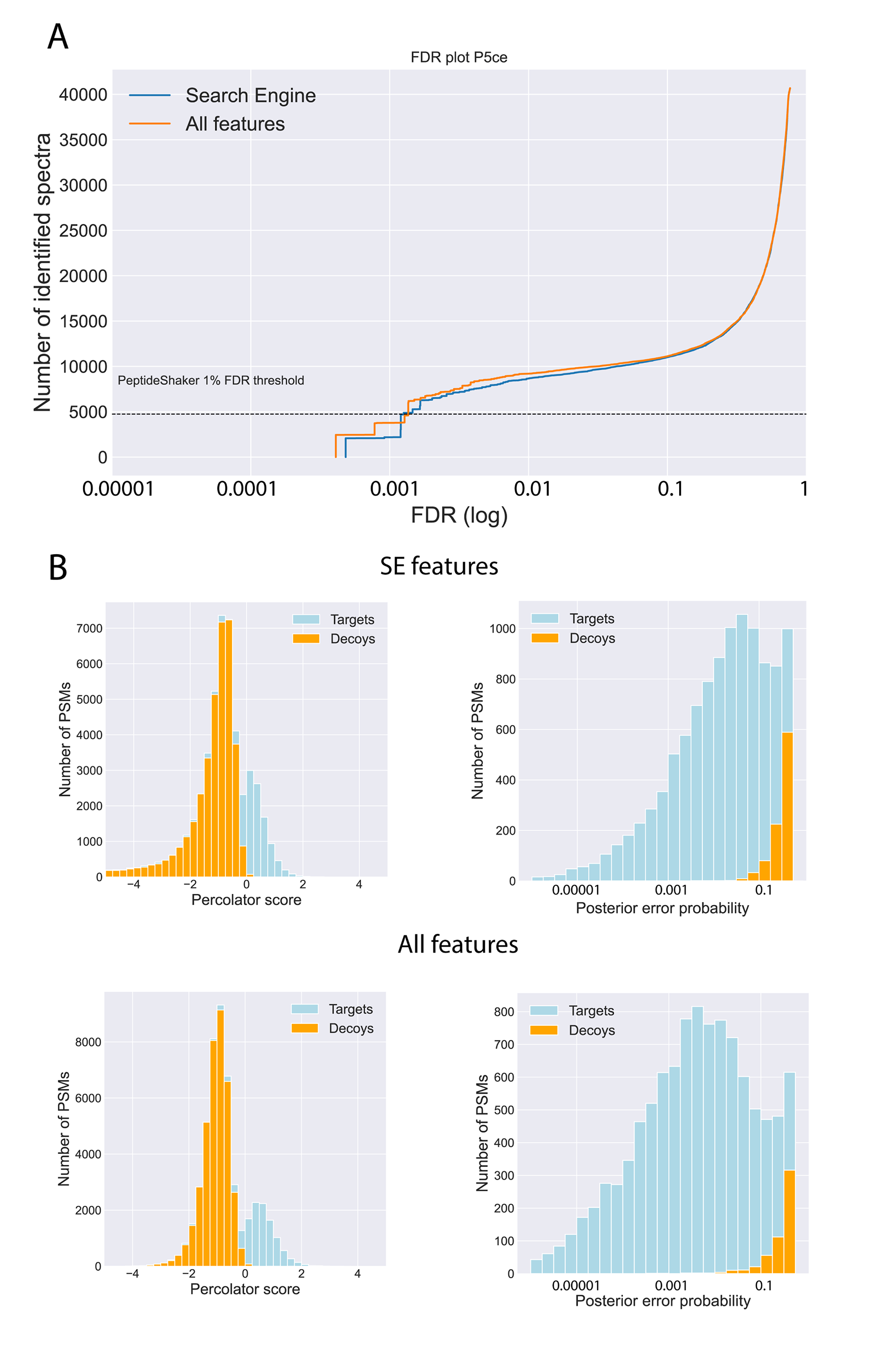

Supplement: Supplementary Figure 6 — Impact of feature prediction on PSM level for cerebellum brain section. [file Image_6.TIF]

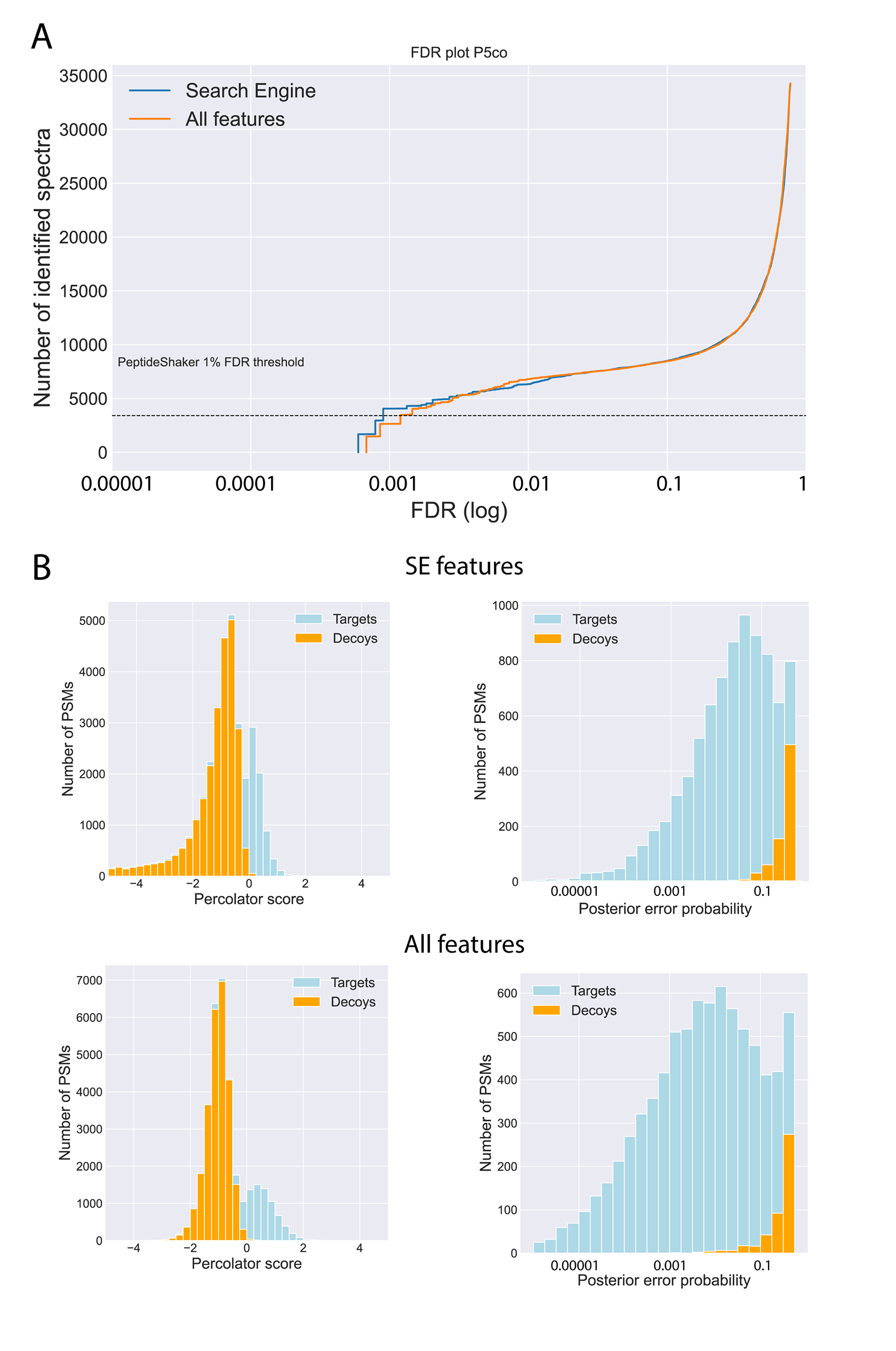

Supplement: Supplementary Figure 7 — Impact of feature prediction on PSM level for cortex brain section. [file Image_7.TIF]

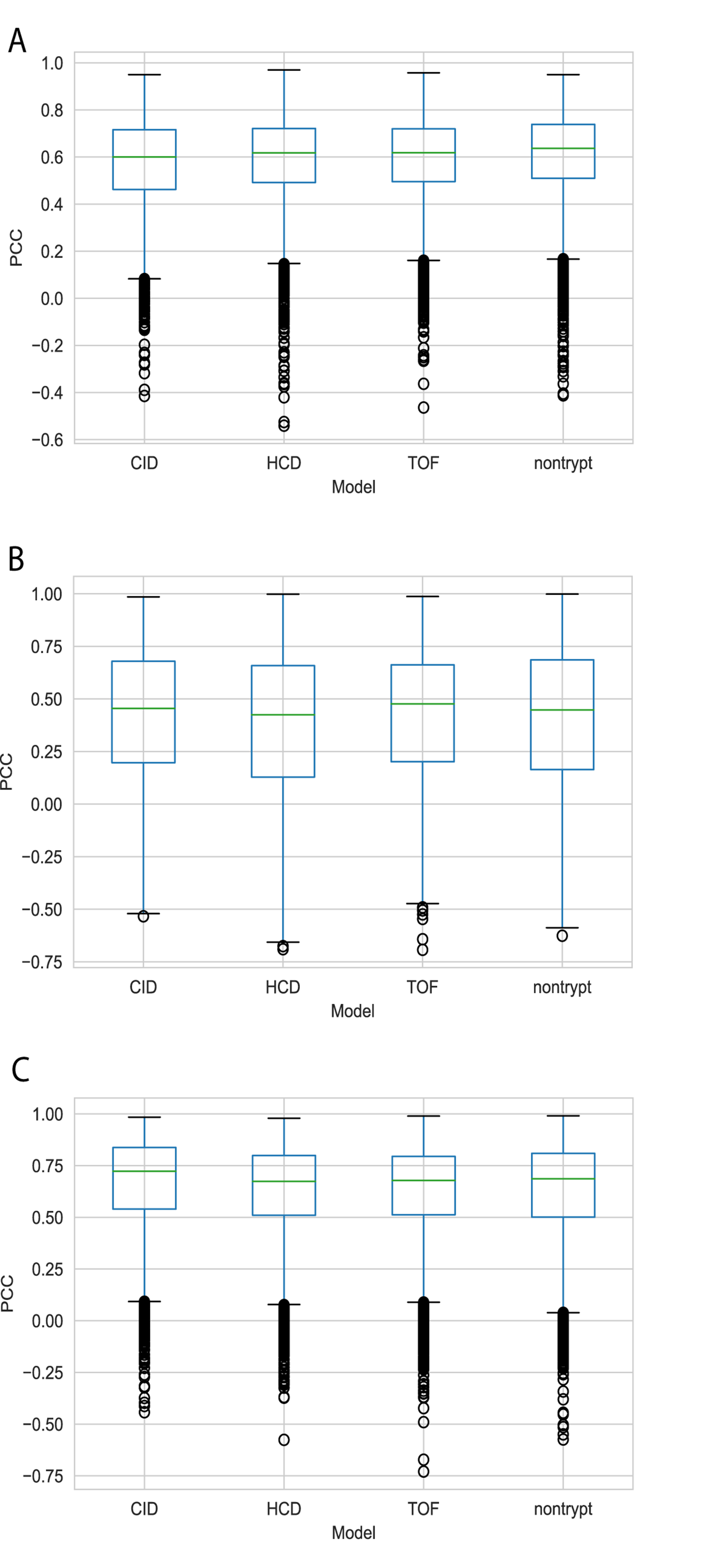

Supplement: Supplementary Figure 8 — Evaluation of the available MS2PIP models of the Pearson Correlation Coefficient for four different models, trained with tryptic peptides of different fragmentations (CID, HCD, TOF) and the non-tryptic model used in this study for b and y-ions combined (A), only y-ions (B) and only b-ions (C). [file Image_8.tif]

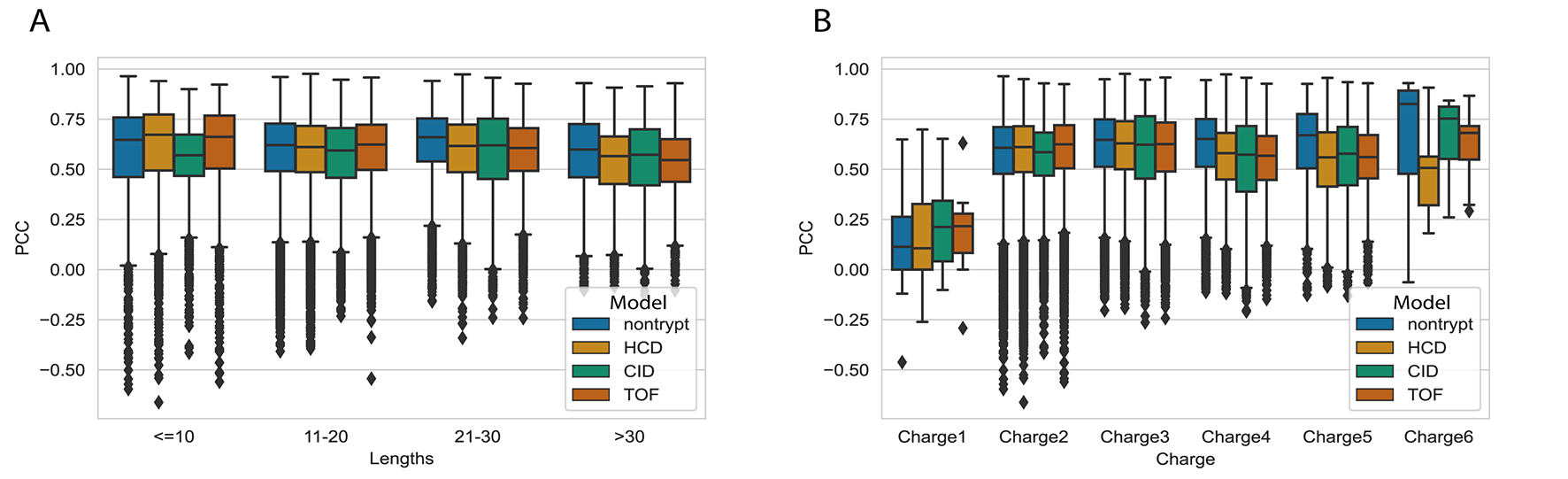

Supplement: Supplementary Figure 9 — Evaluation of peptide of lengths and charge. Boxplots of the Pearson Correlation Coefficient for the different MS2PIP models. [file Image_9.tif]
